# A primer on corollary discharges: neural signals that distinguish ‘Self’ from ‘World’

**DOI:** 10.1038/s44277-026-00063-2

**Published:** 2026-06-11

**Authors:** Thomas J. Whitford, Judith M. Ford

**Affiliations:** 1 School of Psychology, The University of New South Wales, (UNSW Sydney), Sydney, NSW 2052 Australia; 2 Department of Psychiatry, University of California - San Francisco, CA, 94143 US

**Keywords:** Sensory processing, Psychiatric disorders

The brain does not merely react to the world—it also anticipates the consequences of its own actions. Hidden within this predictive machinery lies a signalling motif, corollary discharge, which plays a key role in distinguishing between ‘self’ and ‘world’. A disruption in this mechanism has been implicated in the characteristic symptoms of schizophrenia. But what are corollary discharges, and how do they relate to schizophrenia?

## What are corollary discharges?

Efference copies / corollary discharges are neural signals derived from motor commands that are sent to the sensory processing stream. In other words, they are ‘motor→sensory’ signals, in contrast to the more heavily-studied ‘sensory→motor’ signals required for responses to sensory input.

Efference copies / corollary discharges inform sensory regions about the expected sensory consequences of movements. In doing so, these signals are involved in making the critical distinction between sensations caused by self vs. sensations caused by the world. Accordingly, these signals have been implicated in self-agency: the subjective feeling of being in control of one’s actions.

Corollary discharges are ubiquitous across the animal kingdom [1]. They have been identified in invertebrates with ‘simple’ nervous systems (e.g., nematodes, crickets) where their primary function is sensory filtration and reflex inhibition. They have also been identified in vertebrates with more complex nervous systems (e.g., bats, songbirds, non-human primates, humans), where they have a variety of functions including sensorimotor learning and the temporal coordination of sensory and motor systems.

The distinction between the terms ‘efference copy’ and ‘corollary discharge’ is subtle. In a nutshell, early formulations about ‘motor→sensory’ signalling envisaged a literal copy of the motor signal (i.e., the efference copy) being sent to sensory cortex. In fact, these ‘motor→sensory’ signals can originate at many levels of the motor stream (e.g., motor preparation regions, pre-motor cortex, motor cortex) and can be sent to multiple different levels of the sensory processing stream (e.g., cortex, thalamus, pons, cerebellum, etc.); that is, they are not necessarily exact copies of motor commands that target early levels of the sensory system. Given this, the more general term ‘corollary discharge’ is often used.

## How can corollary discharges be measured?

In non-human animals, corollary discharges have been observed directly from intracellular recordings. A single ‘corollary discharge interneuron’ was found to suppress auditory neurons in the chirping cricket, thereby preventing receptor fatigue [2]. With more sophisticated nervous systems, where the system is more complicated, the action of corollary discharges is typically inferred from their suppressing effects on sensory responses. For example, across species auditory responses are suppressed during vocalization, but not when auditory feedback is distorted. This suggests that corollary discharges don’t just signal *whether* a vocalization has occurred but also contain precise information about the expected sensory properties of the vocalization.

In humans, corollary discharges have been inferred from the sensory responses evoked by various motor actions (e.g., eye movements, finger movements, vocalizations) using a variety of behavioural measures. Perhaps due to being over-learned since infancy, vocalizations produce the most marked suppression of auditory cortical responses, seen in magneto- and electro-encephalography. Most studies have used a variant of the ‘Talking’ paradigm [3]. In this paradigm, participants are asked to repeatedly vocalize a simple syllable (e.g., the syllable ‘ah’) while brain activity is recorded. The brain activity evoked by these vocalizations is compared to the activity evoked by distorted feedback (in real-time), or to the veridical recording played back several minutes later. Auditory cortical activity is reduced when participants talk and hear undistorted feedback, compared to when they either passively listen to the recording, or hear distorted feedback while talking.

**Fig. 1:**
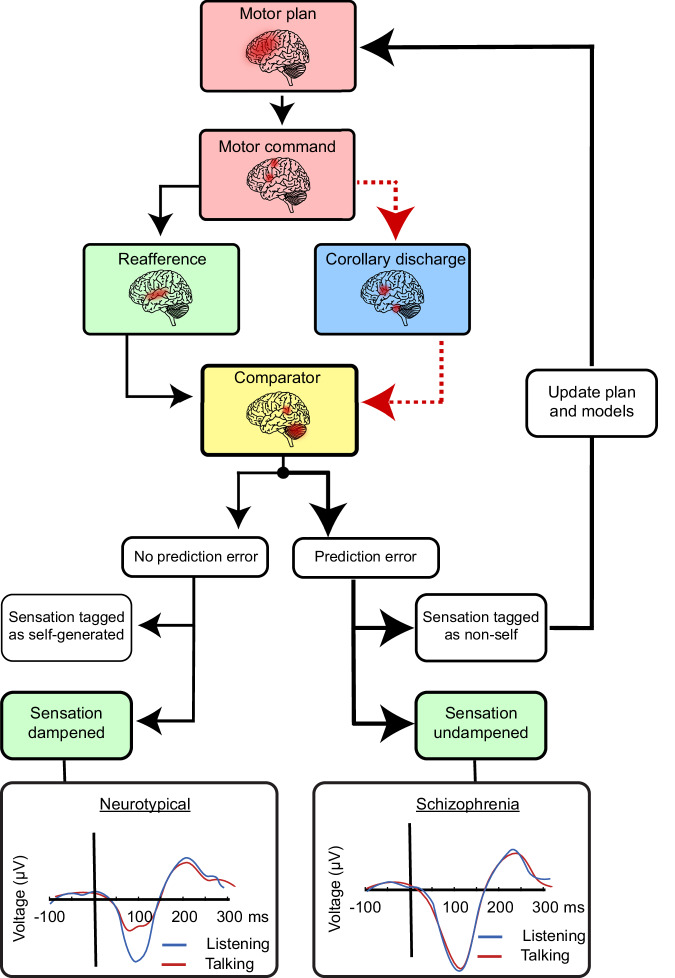
A schematic of the corollary discharge circuit for vocalization, and how it might be disrupted in schizophrenia. Several variants of this circuit have been proposed in the literature. In keeping with the nomenclature used by [1], the motor pathway is shown in pink, and the corollary-discharge system in blue. The motor plan to produce overt speech (likely originating in frontal regions) elicits a motor command (likely originating in ventral premotor cortex [4] and supplementary motor cortex), which causes movements of the articulator organs (mouth, tongue, larynx, etc.), which results in speech-sounds (reafference; shown in green). A transformed version of this motor command (corollary discharge) is used to make predictions about the expected sensory features of the vocalization. The corollary discharge has been linked to both the ventral speech motor cortex [4] and the pons-cerebellar-thalamus circuit. These predictions are compared to the reafferent signals in the comparator module (more speculatively linked to the cerebellum and area Spt; shown in yellow). If the predicted and reafferent sensations match, the sensory response is dampened, as illustrated with the auditory-evoked responses to overt speech vs. passive listening typically exhibited by neurotypical participants. In this case, the vocalization is ‘tagged’ as self-generated. If the signals mismatch, then the sensory response is not dampened, as illustrated with the auditory-evoked responses to overt speech vs. passive listening typically exhibited by schizophrenia participants. This prediction-error is fed forward for further processing, e.g., to update the model, with the aim of minimizing prediction-error for future vocalizations. Schizophrenia has been associated with dysfunctions in the corollary-discharge-related nodes of this circuit (illustrated with red dotted arrows). These corollary-discharge dysfunctions lead to prediction errors, inappropriate updating of motor plans, and sensations being (incorrectly) tagged as not-self-generated (illustrated with the bold black arrows).

While the exact circuitry of the vocalization-related corollary discharge system remains a matter of debate, variants of the circuit described in Fig. 1 have been proposed. These models often include a ‘comparator’ module (speculatively linked to the cerebellum and area Spt) in which the predicted auditory consequences of vocalization (carried in the corollary discharge) are compared to the actual auditory consequences, and the differences between them (i.e., the prediction error) are fed forward for further processing. Intracranial recordings from humans have identified the ventral premotor cortex as the source of the vocalization-induced corollary discharge [4].

## How do corollary discharges relate to schizophrenia?

Corollary discharges play a key role in schizophrenia, from vulnerability to symptoms. In 1978, Feinberg [5] suggested a failure in this mechanism may underpin the breakdown in the distinction between ‘self’ and ‘world’ typical in schizophrenia and psychosis more broadly.

Consistent with this account, there is now a large body of evidence suggesting that people with schizophrenia have corollary-discharge-related abnormalities, including to saccades, smooth pursuit eye-movements, finger movements, and vocalizations. There are over a dozen ‘Talking’ studies showing that patients with schizophrenia and youth at clinical high risk for schizophrenia have less suppression of auditory cortex responses compared to psychologically healthy participants (e.g., [6]). This suggests that psychosis and vulnerability to psychosis have dysfunctional corollary-discharges that may underpin their tendency to misattribute self-generated sensations to external agents (e.g., hallucinations and delusions).

## Are corollary discharges also associated with mental actions?

While traditionally studied in relation to physical actions, recent studies have suggested that corollary discharges may also be associated with certain *mental* actions—like inner speech or thought— and that a dysfunction in these signals may underlie the experience of auditory verbal hallucinations.

It has long been argued that inner speech emerges developmentally from overt vocalization, reflecting an internalized vocalization without articulation. This idea that inner speech is ultimately a ‘kind of action’ which might elicit a corollary discharge is supported by evidence that inner speech is preceded by a signal from motor cortex, like the one that occurs in overt speech.

Consistent with the idea that inner speech elicits a corollary discharge, inner speech is also associated with reduced auditory cortex activity, like the reductions associated with overt vocalization in the Talking task. Most intriguing is that schizophrenia patients show abnormalities in this measure of inner-speaking-induced corollary discharge activity, and that these abnormalities are particularly pronounced in patients with auditory-verbal hallucinations [7].

In summary, corollary discharges are well-established signals seen across the animal kingdom. Corollary discharge deficits likely underpin some psychotic experiences due to their role in distinguishing between ‘self’ and ‘world’. Corollary discharge deficits are associated with vulnerability to psychosis and, as such, these signals are studied in animal models of schizophrenia [8].
